# Short-hairpin RNA-mediated suppression of cortactin may inhibit the migration and invasion abilities of endometrial cancer cells by reducing lamellipodia

**DOI:** 10.22038/IJBMS.2023.67633.14863

**Published:** 2023

**Authors:** Huisi Lin, Yujuan Fan, Zhifu Zhi, Lihong Pang, Dan Sun

**Affiliations:** 1 Department of Gynecology and Obstetrics, The First Affiliated Hospital of Guangxi Medical University, Nanning, China; 2 Department of Gynecology and Obstetrics, University of the Chinese Academy of Sciences, Shenzhen Hospital, Shenzhen, China; 3 Key Laboratory of Early Prevention and Treatment for Regional High-Frequency Tumor (Guangxi Medical University), Ministry of Education, Nanning, China; # These authors contributed eqully to this work

**Keywords:** Cell migration, Cortactin, Endometrial neoplasms, Pseudopodia, Rac1

## Abstract

**Objective(s):**

The prognosis of endometrial cancer (EC) is significantly affected by tumor infiltration and metastasis. Cortactin (CTTN) regulates infiltration and metastasis in other tumors. Studies on the role and mechanism of CTTN in EC are limited and further studies are needed.

**Materials and Methods:**

Quantitative PCR and immunohistochemistry were used to detect Ras-associated C3 botulinum toxin substrate 1 (Rac1) and CTTN in EC and normal tissues. The relationship between the expression of these two genes and their prognostic factors was analyzed. A CTTN-RNAi lentiviral system was constructed and transfected into EC cells. Migration and invasion were evaluated by scratch assay, transwell migration, and invasion assays. Pseudopodia formation was observed by immunofluorescence staining. Western blotting was performed to detect the expression of Rac1.

**Results:**

The expression levels of Rac1 and CTTN in EC tissues were significantly higher than those in normal tissues. In the EC group, Rac1 and CTTN levels were correlated. The protein expression levels of Rac1 and CTTN were related to myometrial invasion and stage. After CTTN knockdown, the migration rate, invasiveness, and migratory ability of EC cells decreased significantly. Lamellipodia was observed to disappear with the appearance of blebs. Rac1 protein expression was decreased after CTTN knockdown.

**Conclusion:**

CTTN may promote the invasion and migration of EC by lamellipodia. This effect may be related to the regulation of Rac1 by CTTN.

## Introduction

Endometrial cancer (EC) is a malignant tumor originating in the endometrium (1). EC is one of the most common gynecological tumors in developed countries (2). Compared with other malignant tumors, their incidence is increasing annually; from 1987 to 2014, there was a 75% increase in cases (3). Although most patients with EC are diagnosed at an early stage and have a good prognosis, 15%–20% of patients are at high risk of distant metastasis, which carries a poor prognosis (4). The tissue-infiltration ability of EC significantly affects patient prognosis (5). Therefore, exploring the invasion and migration mechanisms of EC cells is of great significance to improve the prognosis of patients with this disease.

Cortical actin-binding protein (cortactin) is encoded by the CTTN gene on chromosome 11q13 and has a full length of 45079 bp. CTTN is mainly located in the cell cortex (6). The cell cortex is a plasma membrane-associated part of the actin cytoskeleton, which is primarily responsible for cell mechanics (7). CTTN binds to the Arp2/3 complex to regulate the F-actin network, which in turn regulates actin-based structures such as lamellipodia. Cortactin accumulates in actin-rich lamellipodia and forms membrane folds at the beginning of cell migration (8), which are necessary for the formation of lamellipodia and other cellular structures. CTTN also participates in cell invasion and migration (9), and is frequently amplified in cells with high invasiveness and metastatic potential, such as oral cancer (10), ovarian epithelial cancer (11), and thyroid cancer (12) cells. It has been found to be associated with late tumor stages and lymph node metastasis (10–12). However, few studies have been conducted on CTTN in EC.

Ras-associated C3 botulinum toxin substrate 1 (Rac1), located on human chromosome 7p22, has a total length of 29 kb and contains seven exons. As a key member of the Rho family, Rac1 is an important signaling molecule that regulates cell adhesion and motility. It is highly expressed in gastric cancer, colon cancer, breast cancer, and other malignant tumors (13–15). Under stimulation with platelet-derived growth factor (PDGF), Rac1 initiates lamellipodia formation (16). CTTN induces epithelial-mesenchymal transition (EMT) by participating in the regulation of Rac1 (17). Furthermore, Rac1 activation induces CTTN tyrosine phosphorylation and participates in cytoskeleton remodeling (18). Therefore, we speculated that Rac1 combined with CTTN might affect the invasive and migratory abilities of tumor cells by altering the cytoskeleton. To the best of our knowledge, there have been no studies on the role of CTTN in EC to date, and the mechanism by which Rac1 regulates the effects of CTTN on the invasion and migration of EC is unclear.

In this study, the mRNA and protein expression of Rac1 and CTTN in the tissues was first detected. Quantitative PCR (qPCR) was used to determine the mRNA levels of CTTN in four endometrial cancer cell lines: Ishikawa, AN3CA, HEC-1-A, and HEC-1-B. The HEC-1A and HEC-1B cell lines, which showed the highest expression of CTTN, were selected for further study. After the knockdown of CTTN by CTTN RNA interference (RNAi), changes in tumor cell invasive and migratory abilities, pseudopodia, and Rac1 protein expression were observed, and the invasion and migration mechanisms of EC cells were studied.

## Materials and Methods

***Materials***

Human EC cell lines (Ishikawa, AN3CA, HEC-1-A, and HEC-1-B) were obtained from the National Collection of Authenticated Cell Cultures (Shanghai, China). CTTN-RNAi, HitransGA, and short hairpin RNAi (shRNAi) were purchased from Genechem Biotech Co., Ltd. (Shanghai, China). TRIzol reagent (3101-100) was purchased from Pufei Biotechnology Co., Ltd. (Shanghai, China). M-MLV Reverse Transcriptase (M 1705) was purchased from Promega (Madison, WI, USA). Mouse monoclonal anti-Rac1 antibody (ab33186), rabbit monoclonal anti-cortactin antibody (ab81208), and primary antibody-like anti-F-actin (ab130935) were obtained from Abcam (Cambridge, MA, USA), while the primary antibody anti-Rac1 (bs-4186R) was purchased from Biosynthesis Biotechnology Co. Ltd. (Beijing, China). Secondary antibody goat anti-mouse (A-21424) and McCoy’s 5A medium (16600-082) were purchased from Thermo Fisher Scientific (Massachusetts, USA). HRP-goat anti-rabbit secondary antibody (AS1107) was purchased from ASPEN Biotechnology Co. Ltd (Wuhan, China). Transwell Permeable supports (353097), Corning BioCoat cell culture inserts (354480), Dulbecco’s modified Eagle’s medium (DMEM) (10-013-CVa), and Minimal Essential Medium (MEM) (10-010-CV) were purchased from Corning (NY, USA). Fetal bovine serum (FBS) (VS500T) was purchased from Bovogen (Victoria, Australia). A real-time PCR instrument (LightCycler 480 II) (Roche, Basel, Switzerland) and confocal quantitative image cytometer (CQ1) (Yokogawa, Tokyo, Japan) were used. Tris-EDTA antigen retrieval solution (PR30002) was purchased from Proteintech (Rosemont, IL, USA). A polymer Detection system for immunohistochemical staining (PV-9000) and a DAB kit (ZLI-901) were purchased from ZSGB-Bio (Beijing, China).

***Patient samples***

PASS 15 Power Analysis and Sample Size Software (NCSS, USA) was used to calculate the sample size for the qPCR experiment. The sample size was estimated based on a previous study (19). The means and standard deviations were obtained from previous studies. The software estimated the effect size and indicated the appropriateness of the chosen sample size. A sample size of 7 in the NE group and 8 in the EC group had 90% power to detect a difference between the two groups, with a significance level (alpha) of 0.05 (two-tailed). Group sample sizes of 7 and 8 achieved 95.179% power to detect significant differences in Rac1 expression between the two groups.

Endometrial tissues were obtained from 47 patients with EC (EC group) and 45 patients with a normal endometrium (NE group) at The First Affiliated Hospital of Guangxi Medical University (Nanning, China) between June 2019 and December 2020. None of the patients had received hormone therapy, chemotherapy, or radiotherapy before surgery. All tissues were obtained from hysterectomy specimens. Surgical indications for patients in the NE group were leiomyoma and uterine prolapse. EC staging was assessed based on the International Federation of Gynecology and Obstetrics (FIGO) system (20). All patients provided written informed consent and the study was approved by the Ethics Committee of the First Affiliated Hospital of Guangxi Medical University. The ethics approval number was 2021 (KY-E-309).

***Quantitative PCR***

Total RNA was extracted using the Trizol Reagent. A Moloney murine leukemia virus (M-MLV) kit was used for reverse transcription to obtain single-stranded complementary DNA (cDNA). PCR was performed using cDNA as a template. The primer sequences for CTTN-1 and CTTN-2 were designed based on the CTTN gene (NM_005231). CTTN-1 was used in *in vitro* experiments, and CTTN-2 was used in clinical tissues (Table 1). For the two-step real-time PCR, PCR conditions used were 95 ℃ for 30 sec, followed by 40 cycles of 95 ℃ for 5 sec, and 60 ℃ for 30 sec, and a dissociation program of 95 ℃ for 15 sec, 60 ℃ for 30 sec and 95 ℃ for 15 sec. The gene expression levels were calculated using the 2−ΔΔCT method. The experiment was performed in triplicate.

***Immunohistochemistry(IHC)***

Specimens were fixed in 10% formalin and embedded in paraffin. The experiments were performed using a Polymer Detection system for IHC according to the manufacturer’s instructions. Mouse monoclonal anti-Rac1 (1:200, ab33186) and rabbit monoclonal anti-cortactin (1:200) antibodies were added to the sections. The positive staining percentage was graded as follows:0, no positive cells; 1, 25% or fewer positive cells; 2, 26–50% positive cells; 3, 51–75% positive cells; and 4, 76% or more positive cells. Staining intensity was graded as follows:0, no intensity; 1, weak intensity; 2, moderate intensity; and 3, strong intensity (21). The total score was calculated as the product of these two scores. A total score of less than 6 indicated low expression and a total score greater than or equal to 6 indicated high expression.

***Construction of a lentiviral system***

The complete CTTN sequence (NM_005231) was obtained from the GeneBank database. Next, shRNA interference target sequences were identified using an Invitrogen BLOCK-iT RNAi Designer (Invitrogen, Carlsbad, CA, USA). Using the basic local alignment search tool (BLAST) for comparison with the genome database, homologous coding sequences were excluded to determine three shRNA interference target sequences as follows:

CTTN-KD1 (5′-cgGCAAATACGGTATCGACAA-3′); 

CTTN-KD2 (5′-gaAAGACTACTCCAGTGGTTT-3′); and 

CTTN-KD3 (5′-caCGAATATCAGTCGAAACTT-3′). 

The plasmids were synthesized by Shanghai Genechem Biotech Co. Ltd (Shanghai, China).

***Cell transfection and grouping***

HEC-1-A and HEC-1-B cells were passaged, inoculated into 6-well plates at 2 × 10^5^ cells/well, and cultured for 2 days. Each cell line was divided into experimental groups (KD1, KD2, and KD3) and a control group (CON). The KD1, KD2, and KD3 groups were transduced with CTTN-KD1, CTTN-KD2, and CTTN-KD3 lentiviral vector plasmids, respectively, and the CON groups were transduced with an empty vector (lentiviral vector without siRNA), for 24 hr in 40 µl 1X HitransG A. Cells were incubated in McCoy’s 5A medium (HEC-1-A) or MEM (HEC-1-B) containing 10% FBS for 72 hr. The relative mRNA expression of CTTN was assessed by quantitative PCR to determine the transfection efficiency in EC cells, which were subjected to cell migration, invasion, scratch assays, immunofluorescence staining, and western blot analysis. 

***Transwell migration and invasion assay***

For the cell migration assay, serum-starved transduced cells (HEC-1-A 2×10^5^ cells/ well, HEC-1-B 1×10^5^ cells/well) were suspended and added to the upper chambers of 24-well Transwell plates with 8.0-μm pores. Full-serum medium containing 10% FBS was added to the lower chamber as a chemoattractant. After incubation at 37 °C with 5% CO_2_ for 24 hr, the cells attached to the lower surface of the membrane were fixed with methanol and stained with 0.1% crystal violet. The migratory cells were then observed under a microscope (magnification ×200, XDS-100; Shanghai Caikon Optical Instrument Co. Ltd., Shanghai, China), and the number of invading cells in nine randomly selected fields was counted and averaged. The cell invasion assay was performed in the same manner as the cell migration assay, except that the upper chambers of the 24-well Transwell plates were pre-coated with a Matrigel membrane (Corning, USA).

***Scratch assays***

After 72 hr of transfection, the cells in each group were cultured to the logarithmic growth phase, and the cell density was adjusted to 1×10^6 ^cells/ml in a 96-well plate until the cells reached more than 90% confluence. The cells were then seeded into a 96-well plate. After adhering to the wall, cells with good growth and uniform density were used for the scratch assay. To perform the scratch assay, a scratch instrument was placed at the center of the upper end of a 96-well plate and was gently pushed upward to form scratches. Residual cells in the scratch area were washed away using phosphate-buffered saline (PBS). After culture, cell migration was detected using a Celigo scanning plate at 0 and 48 hr (for HEC-1-A cells) and 0 and 24 hr (for HEC-1-B cells) after scratching. The cell areas at the time of detection were analyzed, and cell mobility was calculated. The migration area was calculated as the cell area at 24 hr/48 hr minus the cell area at 0 hr. The mobility was calculated as the migration area/1-cell area at 0 hr. Each group contained three compound wells.

***Western blot***

After 72 hr of transfection, the total proteins of cells in each group were extracted, and sodium dodecyl sulfate–polyacrylamide gel electrophoresis (SDS-PAGE) was performed. After transmembrane transfer, skim milk powder was added to the cells, which were then blocked for 1 hr. The first antibody (rabbit anti-human Rac1 antibody, 1:500,bs-4186R) was then added for incubation overnight, following which the secondary antibody (1:10000) was added for 1-hr incubation, with β-actin serving as the internal control. The band intensity was quantified using ImageJ software. The target protein level was expressed as the grey value of the target protein band/β-actin band. The experiment was repeated thrice.

***Immunofluorescence staining***

After transfection, HEC-1-A and HEC-1-B cells were cultured to the logarithmic phase, and a 1×10^4^ single-cell suspension was prepared and inoculated into a 96-well glass-bottom plate (a special laser confocal culture plate). The plates were then placed in an incubator until the cells formed a monolayer. After rinsing twice with PBS, the cells were fixed with 4% paraformaldehyde for 10 min and rinsed again with PBS for 5 min. Permeabilization was performed using 0.2% Triton for 5 min, after which cells were rinsed twice with PBS for 5 min each time. A blocking solution (PBS containing 5% BSA and 10% sheep serum) was added, and the plate was placed in a wet box at room temperature for 30 min. F-actin antibody (1:200) was added, and the plate was refrigerated at 4 °C overnight before being rinsed thrice in PBS for 5 min each time. Fluorescein isothiocyanate (FITC)-labeled goat anti-mouse fluorescent IgG secondary antibody (1:400) was added, and the plate was incubated in the dark at 37 °C for 45 min. The secondary antibody was discarded, and the cells were rinsed thrice with PBS for 5 min each time and then once with distilled water. The cells were then incubated with DAPI (4′,6-diamidino-2-phenylindole) at room temperature for 10 to 15 min, before being rinsed twice with PBS, for 5 min each time. Finally, 100 µl PBS was added to each well for preservation and observation.

***Laser confocal microscopy***

A quantitative image cytometer (CQ1, Yokogawa, Japan) was used to obtain the best tomographic images of the cells with excitation filter wavelengths of 405, 488, and 561 nm. Each of the four quadrants of the culture dish was observed, and cell morphology was recorded.

***Statistical analysis***

SPSS 22.0 (IBM, Armonk, NY, USA) was used for statistical analysis. The t-test was used to analyze the measurement data. One-way analysis of variance (ANOVA) was used to analyze the data. Correlation analysis was performed using the Pearson or Spearman correlation analysis. A *P*-value<0.05 was considered statistically significant.

## Results

***Clinical baseline characteristics of the EC and NE groups***

Table 2 shows the baseline clinical parameters. The average age of patients in the EC group was 55.85 ± 8.58 years old, and that in the NE group was 53.24 ± 8.32 years old (*P=*0.143). There were no differences in menopausal age (*P=*0.135), menopause (*P=*0.097), gravidity (*P=*0.170), parity (*P=*0.281), systolic pressure (*P=*0.253), diastolic pressure (*P=*0.348), and BMI (*P=*0.321) between the two groups. The results showed that the two groups were comparable.

**Figure 1:**
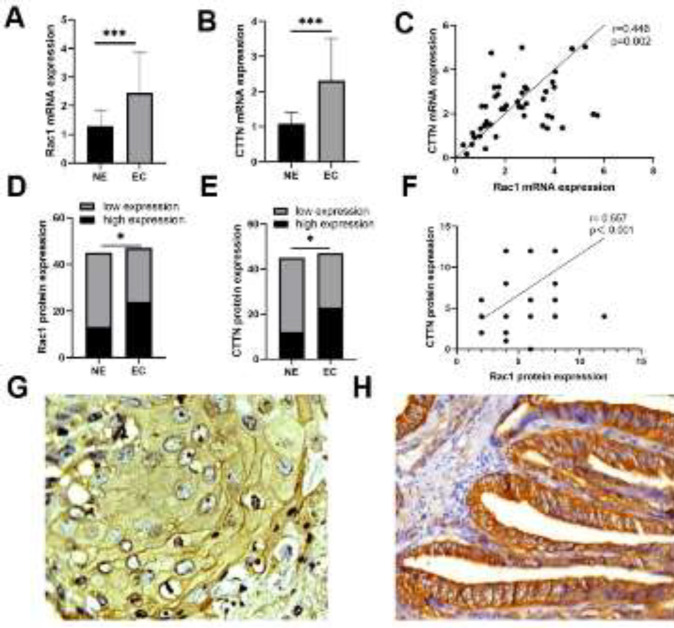
mRNA and protein expression of Rac1 and CTTN in the two groups of NE and EC

Figure 1 shows the mRNA and protein expressions of Rac1 and CTTN in the normal endometrium and type I EC using qPCR and IHC. The relative mRNA expression levels of Rac1 and CTTN were higher in the EC group than those in the NE group (Figure 1A, 1 B). In the NE group, 13 cases with high expression of the Rac1 protein were fewer than 32 cases with low expression of the Rac1 protein (28.89% vs 71.11%), whereas, in the EC group, 24 cases with high expression of the Rac1 protein were slightly higher than 23 cases with low expression (51.06% vs 48.94%, *P*<0.05, Figure 1D). In the NE group, 12 cases with high expression of the CTTN protein were fewer than 33 cases with low expression (26.67% vs 73.33%), whereas, in the EC group, 23 cases with high expression of CTTN were slightly lower than 24 cases with low expression (48.94% vs 51.06%, *P*<0.05, Figure 1E). There was a positive correlation between the mRNA expression of Rac1 and CTTN expression in the EC group (r=0.446, *P*=0.002; Figure 1C). Similarly, there was a positive correlation between Rac1 and CTTN protein expression in the EC group (r=0.557, *P*<0.001; Figure 1F). IHC showed that the Rac1 protein was localized in the cell membrane (Figure 1G) and the CTTN protein was localized in the cytoplasm (Figure 1H). 

We further analyzed the relationship between Rac1 and CTTN protein expression and clinicopathological features of EC. Between the high and low expression groups of Rac1 protein, there were significant differences in LVSI (*P*=0.008), depth of myometrial invasion (*P*=0.020), and FIGO stage (*P*=0.003). In the group with high expression of CTTN, there were significant differences in the depth of myometrial invasion (*P*=0.013) and FIGO stage (*P*=0.001). In the high expression groups of Rac1 and CTTN proteins, there were more patients with myometrial invasion >1/2 and those at FIGO stages III-IV. There were more LVSI-positive patients in the group with high Rac1 expression (Table 3).

***Expression of CTTN mRNA in four EC cell lines***

**Figure 2:**
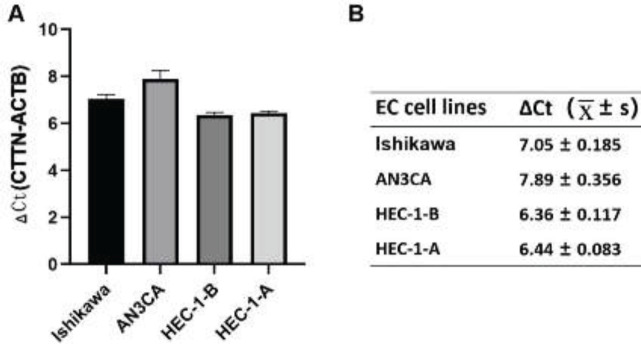
Expression of CTTN mRNA in Ishikawa, AN3CA, HEC-1-B, and HEC-1-A cell lines

To screen EC cell lines with high CTTN expression, we estimated the mRNA expression levels of CTTN in the Ishikawa, AN3CA, HEC-1-B, and HEC-1-A cell lines. CTTN was found to be highly expressed in Ishikawa, AN3CA, HEC-1-B, and HEC-1-A cells (Figure 2A). CTTN expression was higher in the HEC-1-B and HEC-1-A cell lines (6.36±0.117 and 6.44±0.083, respectively) than in the Ishikawa (7.05±0.185) and AN3CA (7.89±0.356) cell lines (Figure 2B). Therefore, HEC-1-A and HEC-1-B cells were selected for follow-up studies.

***Effects of RNAi on CTTN gene expression in HEC-1-A and HEC-1-B cells***

**Figure 3:**
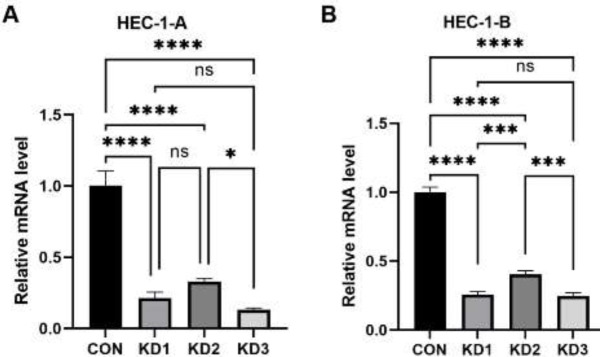
Expression of CTTN mRNA in EC cells transfected with three

Compared with that in the CON group (1.003±0.103), CTTN gene expression in HEC-1-A cells was significantly decreased after knockdown using three different CTTN-RNAi lentiviral systems (*P*<0.001, Figure 3A). The mRNA expression levels of CTTN in the KD1, KD2, and KD3 groups were 0.212±0.043, 0.330±0.021, and 0.131±0.014, respectively. CTTN gene knockdown efficiencies in the KD1, KD2, and KD3 groups were 78.8%, 67%, and 86.9%, respectively. The knockdown efficiency was higher in the KD1 and KD3 groups than in the KD2 group; therefore, the KD1 and KD3 groups were selected for follow-up studies.

Compared with that in the CON group (1.000±0.036), CTTN gene expression in HEC-1-B cells decreased significantly after knockdown (*P*<0.001, Figure 3B). The mRNA expression levels of CTTN in the KD1, KD2, and KD3 groups were 0.258±0.022, 0.405±0.026, and 0.246±0.023, respectively. CTTN gene knockdown efficiencies in the KD1, KD2, and KD3 groups were 74.2%, 59.5%, and 75.4%, respectively. The knockdown efficiency was higher in the KD1 and KD3 groups than in the KD2 group; therefore, the KD1 and KD3 groups were selected for follow-up studies.

**Figure 4:**
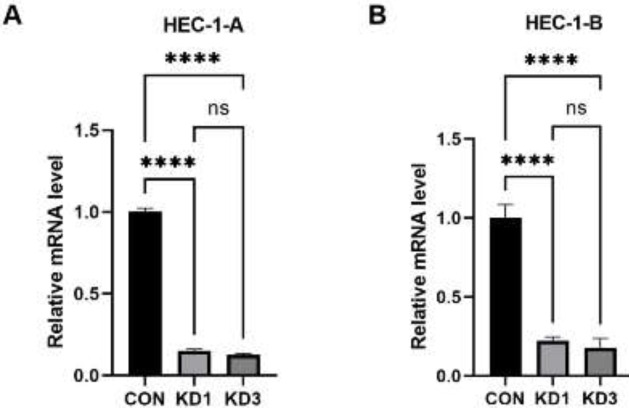
Expression of CTTN mRNA in EC cells transfected with two selected

We verified the knockdown efficiency before conducting follow-up tests. In HEC-1-A cells, the CTTN knockdown efficiencies in the KD1 and KD3 groups were 84.8% and 87.5%, respectively (Figure 4A). In HEC-1-B cells, the CTTN knockdown efficiencies in the KD1 and KD3 groups were 77.6% and 82.1%, respectively (Figure 4B).

***Effects of CTTN knockdown on the migratory ability of HEC-1-A and HEC-1-B cells***

**Figure 5:**
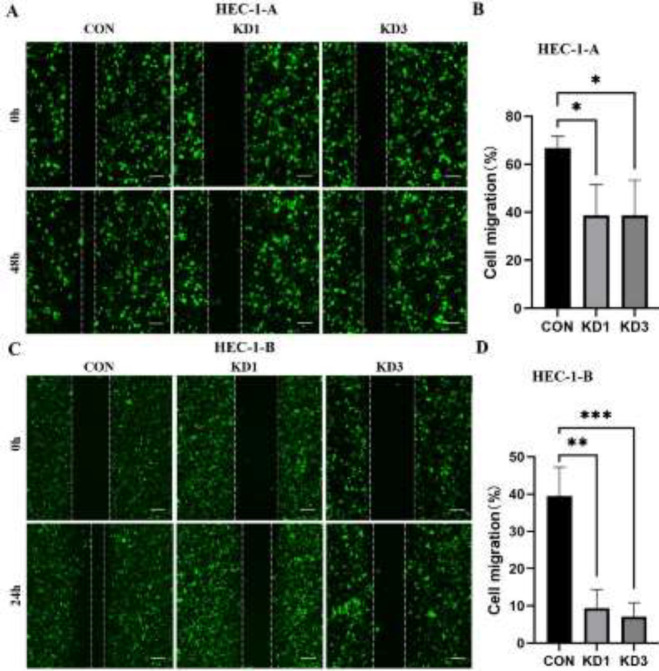
Scratch assay to determine the migration ability of EC cells in each group of HEC-1-A and HEC-1-B

The migration rates of HEC-1-A cells were 38.72±12.87% in the KD1 group and 38.75±14.60% in the KD3 group, compared with 66.86±4.83% in the CON group (Figure 5B). The migration rates of HEC-1-B cells in the KD1 group and the KD3 group were 9.40±5.05% and 7.11±3.74%, respectively, compared with 39.47±7.76% in the CON group (*P*<0.001, Figure 5D).

**Figure 6:**
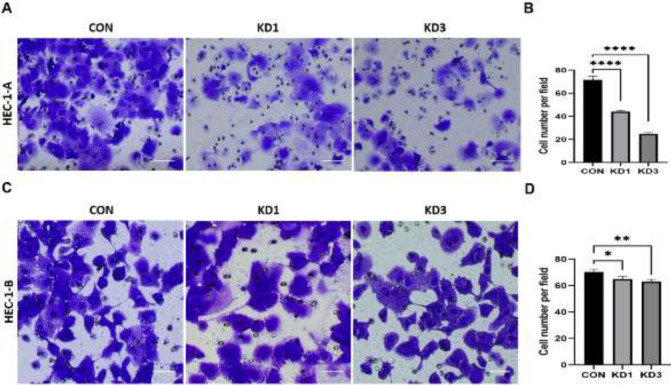
Effects of CTTN knockdown on the migration ability of EC cells in HEC-1-A and HEC-1-B

After CTTN-RNAi transfection, the number of HEC-1-A cells that had migrated through the membrane was 72±2.90 in the CON group, compared with 44±0.62 in the KD1 group (*P*<0.0001) and 25±1.20 in the KD3 group (*P*<0.0001, Figure 6B). The number of HEC-1-B cells that had migrated through the membrane was 71±2.27 in the CON group, compared with 65±2.07 in the KD1 group (*P=0.*010) and 63±2.09 in the KD3 group (*P=0.*002, Figure 6D). For both cell lines, the number of migrating cells was significantly decreased in the KD1 and KD3 groups compared to that in the CON group.

***Effects of CTTN knockdown on the invasive ability of HEC-1-A and HEC-1-B cells***

**Figure 7:**
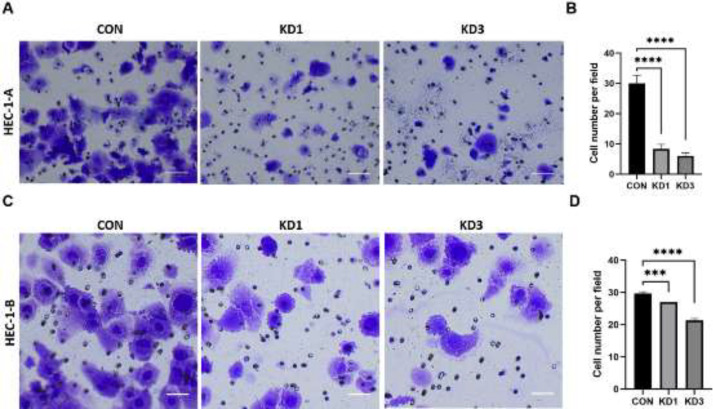
Effects of CTTN knockdown on the invasion ability of EC cells in HEC-1-A and HEC-1-B

After CTTN-RNAi transfection, the number of HEC-1-A cells invading the membrane was 30±2.58 in the CON group, compared with 8±1.25 in the KD1 group (*P*<0.0001) and 5±0.88 in the KD3 group (*P*<0.0001, Figure 7B). The number of HEC-1-B cells invading the membrane was 30±0.67 in the CON group, compared with 26 ±0.36 in the KD1 group (*P*<0.001) and 22 ±1.09 in the KD3 group (*P*<0.0001, Figure 7D). For both cell lines, the number of invasive cells was significantly lower in the KD1 and KD3 groups than in the CON group.

***Effects of CTTN knockdown on pseudopodia in HEC-1-A and HEC-1-B cells***

**Figure 8:**
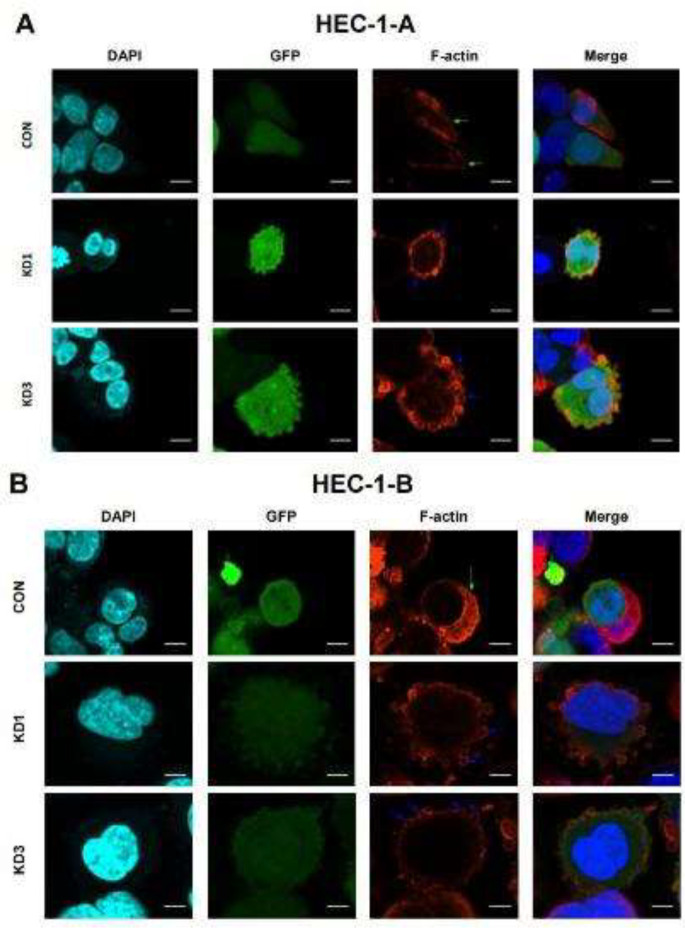
Effects of CTTN knockdown on pseudopodia in EC cells

After CTTN knockdown, the observation of HEC-1-A and HEC-1-B cells under a confocal laser scanning microscope showed that lamellipodia were observed to disappear with the appearance of blebs (Figure 8A, 8B). After transfection of CTTN-RNAi into HEC-1-A and HEC-1-B cells, pseudopodia were observed by F-actin staining and confocal laser microscopy. At the edge of the cell membrane, the blebs were blister-like and the lamellipodia were broad and flaky. In both cell lines, after the knockdown, there were higher numbers of blebs in the KD1 and KD3 groups than in the CON group, whereas the number of lamellipodia showed the opposite trend.

***Effects of CTTN knockdown on Rac1 protein expression in HEC-1-A and HEC-1-B cells***

**Figure 9:**
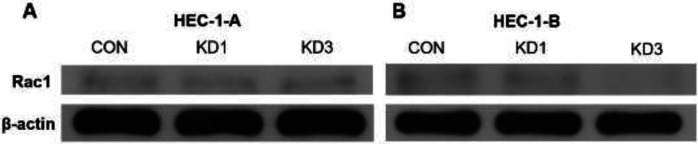
Effects of CTTN knockdown on Rac1 protein expression in EC cells

After CTTN knockdown in HEC-1-A cells, the band density of the Rac1 protein was lower in the KD1 (0.086) and KD3 (0.096) groups than in the CON group (0.116) (Figure 9A). Similarly, in HEC-1-B cells, the band density of the Rac1 protein in the KD1 (0.075) and KD3 (0.029) groups was lower than that in the CON group (0.093) following CTTN knockdown (Figure 9B). Compared to the CON group in both cell lines, the expression of Rac1 decreased after CTTN knockdown.

**Table 1:** PCR primers and sequences for qPCR analysis

Gene	Primer sequence (5’ 3’)
f-hRac1 r-hRac1 f-hCTTN-1 r-hCTTN-1 f-hCTTN-2 r-hCTTN-2 f-hACTB r-hACTB	ATGTCCGTGCAAAGTGGTATC CTCGGATCGCTTCGTCAAACA CTACGATGAGTACGAGAACG ATGATGTCATCAGGGTCAA AGACCGACCCTGATTTTGTGA GTGCTTGGAAAGTTTCGACTGA GCGTGACATTAAGGAGAAGC CCACGTCACACTTCATGATGG

mRNA expression level of Rac1 (A) and CTTN (B) in NE and EC groups. (C) The correlation of Rac1 and CTTN mRNA expression in the EC group. The cases of low- and high-protein expression of Rac1 (D) and CTTN (E) in NE and EC groups. (F) The correlation of Rac1 and CTTN protein expression in the EC group. IHC staining of Rac1 (G) and CTTN (H) in EC tissues. *P<0.05, ***P<0.001. ×200 magnification.

**Table 2:** Basic characteristics of the clinical parameters of patients

**Variables**	**EC group (n=47)**	**NE group (n=45)**	**t/χ** ^2^	***P***
Age (years)	55.85±8.58	53.24±8.32	1.478	0.143
Gravidity			3.667	0.170
0	2（4%）	6（13%）		
1	5（11%）	8（18%）		
≥2	40（85%）	31（69%）		
Parity			1.162	0.281
0	3（6%）	7（16%）		
≥1	44（94%）	38（84%）		
Menopausal age (years)	14.45±2.03	13.91±1.31	1.51	0.135
Menopause			2.755	0.097
no	17（36%）	24（53%）		
yes	30（64%）	21（47%）		
BMI（kg/m^2^）	24.98±5.78	24.01±3.05	0.998	0.321
Blood pressure (mmHg)				
Systolic pressure	130.38±15.15	126.71±15.43	1.151	0.253
Diastolic pressure	77.26±7.65	79.13±11.06	-0.944	0.348

**Table 3:** Pathological parameters of EC patients in high and low expression groups of Rac1 and CTTN protein expression

**Pathological** **parameters**	**n**	**Rac1 expression**		***P***	**CTTN expression**		***P***
		**High** **达**	**Low**		**High**	**Low**	
Pelvic lymph node invasion							
Positive	6	5	1	0.209	4	2	0.622
Negative	41	19	22		19	22	
LVSI							
Positive	8	8	0	0.008	6	2	0.218
Negative	39	16	23		17	22	
Depth of myometrial invasion							
＜1/2	36	15	21	0.020	14	22	0.013
≥1/2	11	9	2		9	2	
FIGO stage							
I-II	36	14	22	0.003	13	23	0.001
III-IV	11	10	1		10	1	
Histologic grade							
1	21	11	10	0.871	8	13	0.181
2-3	26	13	13		15	11	

Rac1, Ras-related C3 botulinum toxin substrate 1; CTTN, Cortactin; FIGO, International Federation of gynecology and obstetrics; LVSI, lymph vascular space invasion

mRNA expression levels of CTTN in Ishikawa, AN3CA, HEC-1-B, and HEC-1-A cell lines were shown in bar chart(A) and three-line table(B). ACTB was selected as the internal reference gene. Δ Ct= target gene Ct value-internal reference gene Ct value. CTTN: Cortactin; Ishikawa, AN3CA, HEC-1-A, and HEC-1-B were human EC cell lines.

CTTN-RNAi lentiviral systems for screening

(A) Expression of CTTN mRNA in HEC-1-A. (B) Expression of CTTN mRNA in HEC-1-B. The KD1, KD2, and KD3 groups were transduced with CTTN-KD1, CTTN-KD2, and CTTN-KD3 lentiviral vector plasmids, respectively, and the CON groups were transduced with an empty vector (lentiviral vector without siRNA). ns = *P*>0.05, *P<0.05, ***P<0.001, and ****P<0.0001.

CTTN: Cortactin

CTTN-RNAi lentiviral systems

(A) Expression of CTTN mRNA in HEC-1-A. (B) Expression of CTTN mRNA in HEC-1-B. The KD1 and KD3 groups were transduced with CTTN-KD1 and CTTN-KD3 lentiviral vector plasmids, respectively, and the CON groups were transduced with an empty vector (lentiviral vector without siRNA). ns = *P*>0.05, ****P<0.0001.

CTTN: Cortactin

(A) The cell migration in HEC-1-A was analyzed. (B) Data are expressed as means±SD of HEC-1-A.(C) The cell migration in HEC-1-B was analyzed. (D) Data are expressed as means±SD of HEC-1-B.*P<0.05, **P<0.01, and ***P<0.001. All scale bars: 200 µm.

(A) The cell migration in HEC-1-A was analyzed. (B) Data are expressed as means±SD of HEC-1-A.(C) The cell migration in HEC-1-B was analyzed. (D)Data are expressed as means±SD of HEC-1-B. *P<0.05, **P<0.01, and ****P<0.0001. All scale bars: 150 µm.

(A) Cell invasion in HEC-1-A was analyzed. (B) Data are expressed as means±SD of HEC-1-A. (C) Cell invasion in HEC-1-B was analyzed. (D) Data are expressed as means±SD of HEC-1-B. ***P <0.001, ****P <0.0001. All scale bars: 150 µm.

Representative images of HEC-1-A cells and HEC-1-B cells were shown in (A) and (B), respectively. blue,nuclei;red, F-actin;green,GFP.DAPI: 4’, 6-diamidino-2-phenyl indole; GFP: green fluorescent protein. Green arrow indicates lamellipodia. Blue arrow indicates blebs. All scale bars: 10 µm.

CTTN: Cortactin

Rac1 protein expression was measured by western blot in HEC-1-A(A) and HEC-1-B(B) cells and calculated according to the ratio of the gray value of the Rac1 protein band to that of the β-actin band.

CTTN: Cortactin

## Discussion

Rac1 and CTTN play major roles in promoting epithelial tumor metastasis, and the activation of these two genes has been reported in ovarian, breast, and colon cancers (11, 14, 15). First, through the examination of clinical tissues, we found that high expression of Rac1 and CTTN was related to poor prognosis of EC. Our preliminary conclusions are consistent with those of the above studies. Therefore, we investigated this *in vitro* mechanism.

CTTN is involved in the invasion and metastasis of tumor cells (17). It is transferred from the perinuclear cytoplasm to the cell periphery, where it first activates Rac1 and Cdc42 (21) and then the downstream F-actin nucleation-promoting factor before finally activating the Arp2/3 complex (23). CTTN can also directly bind to the Arp2/3 complex to stimulate actin nucleation, resulting in dynamic changes in the cytoskeleton and promotion of lamellipodia formation, which are involved in cellular movement (17). Our study found that the invasive and migratory abilities of EC cells decreased significantly after CTTN knockdown, suggesting that CTTN might be involved in EC cell invasion and migration.

The plasma membrane extends forward at the front edge of the cell to initiate cell movement (24). Four different types of protuberances are involved in plasma membrane extension: lamellipodia, filopodia, invadopodia, and blebs (25). Pseudopodia include lamellipodia, filopodia, and invadopodia, which are the main executors of tumor cell movement (26), and their number is related to the migratory ability of malignant tumor cells (27). Increased AMP-activated protein kinase (AMPK) activity and down-regulation of the Jun N-terminal kinase (JNK) pathway can reduce lamellipodia formation and cell migratory ability (9).

Lamellipodia are primarily responsible for cell invasion and long-distance migration (28). Microfilaments are the main components of pseudopodia, which are assembled by the actin monomer G-actin. The formed fiber bundle, a double-stranded helix called F-actin, is implicated in the dynamic changes in polymerization and depolymerization, which can drive pseudopodia extension and movement (29). Blebs were first described in 1919 as transparent cellular blebbing (30) of 2 μm to 15 μm in diameter. They were later described as smooth circular extensions of the plasma membrane that contracted from the cytoplasm to their initial position (31). Blebs promote directional cell migration during development (32). Vesicle-based migration has also been observed in cancer cells (33). High cell tension is beneficial for bleb growth, whereas activation of Arp2/3 reduces tension and facilitates lamellipodia formation (34). Therefore, lamellipodia formation can be blocked by inhibiting the function of the wave regulatory complex (WRC) or Arp2/3 complex, which in turn promotes bleb formation (35). The ability of cancer cells to switch between different migration modes enables their adaptation to different environments, which helps to maintain their high invasiveness (36). In this study, immunofluorescence staining revealed that lamellipodia was observed to disappear with the appearance of blebs, and cell migratory and invasive abilities decreased after CTTN knockdown, consistent with the findings of a previous study(36). Our results suggest that CTTN affects the migratory and invasive abilities of EC cells by regulating lamellipodia. However, the mechanism by which CTTN regulates EC movement needs to be further explored.

Rac1 is the node at which multiple signaling pathways such as tumor proliferation, invasion, and movement converge (37). The activation of Rac1 increases tumor invasiveness (38). Rac1 regulates lamellipodia formation (39) and migration and invasion of tumor cells (40). Migration and invasion inhibitory proteins (MIIP) can block the Rac1 signal transduction pathway and inhibit the migration of EC cells by directly binding to the downstream effector molecule, PAK1 (41). PAK1 controls the cytoskeleton, particularly lamellipodia formation, primarily by regulating the structure of polymeric actin (42). In the present study, we found that the expression of Rac1 decreased after CTTN knockdown, suggesting that CTTN might affect the expression of Rac1. Deletion of CTTN has been found to reduce Rac1 activity, while transfection of cells with active Rac1 can preserve the phenotype, indicating that CTTN may be a branch sensor that activates Rac1 (43). Interestingly, some studies have demonstrated that CTTN is a downstream effector of the Rac1 pathway (44). Therefore, some researchers have proposed that Rac1 and CTTN form positive and negative feedback networks (45). We found that Rac1 protein expression decreased in the experimental groups after CTTN knockdown. Therefore, CTTN may affect cell movement by regulating Rac1. However, the specific regulatory mechanisms need to be explored in further studies.

## Conclusion

The study found that CTTN may improve the invasion and migration abilities of EC cells by lamellipodia. This phenomenon may be related to the regulation of Rac1 by CTTN, but the specific regulation mechanism needs further study.

## Authors’ Contributions

D S designed and administered the experiments and edited the manuscript. LH P supervised the data analysis and edited the manuscript. HS L performed the experiments, analyzed and interpreted the data, and wrote the manuscript. YJ F provided technical support, and designed and analyzed the experiments. ZF Z handled the picture and article format. All authors contributed exhaustively to the realization of this study.

## Availability of Data and Materials

Upon reasonable request, all data in this published article are available from the corresponding author.

## Ethical Approval

This clinical study was approved by the Medical Ethics Committee of First Affiliated Hospital of Guangxi Medical University, China. 

## Statement

This preprint has been presented as a preprint in Research Square according to the following link (https://www.researchsquare.com/article/rs-1438332/v1).

## Conflicts of Interest

The authors have no conflicts of interest to declare.
